# Artificial Intelligence in Embryo Selection: Current Approaches and Clinical Implications

**DOI:** 10.3390/bioengineering13060688

**Published:** 2026-06-16

**Authors:** Lucia Maresca, Antonio D’Amato, Camilla Coianiz, Alessandra Cavalieri, Erica Vella, Francesco Gebbia, Lorena Bori, Marcos Meseguer

**Affiliations:** 1 IVIRMA Global Research Alliance, IVI Roma, 00161 Rome, Italy; lucia.maresca@ivirma.com (L.M.); camilla.coianiz@ivirma.com (C.C.); alessandra.cavalieri@ivirma.com (A.C.); erica.vella@ivirma.com (E.V.); francesco.gebbia@ivirma.com (F.G.); 2 Department of Medicine, University of Valencia, 46010 Valencia, Spain; 3 IVIRMA Global Research Alliance, IVI Valencia, IVI Foundation, Instituto de Investigación Sanitaria La Fe, 46026 Valencia, Spain; lorena.bori@ivirma.com (L.B.); marcos.meseguer@ivirma.com (M.M.)

**Keywords:** artificial intelligence, embryo selection, in vitro fertilization, machine learning, deep learning, time-lapse imaging, morphokinetics

## Abstract

Embryo selection remains one of the main unresolved challenges in in vitro fertilization, despite major advances in assisted reproductive technologies. Conventional assessment is still largely based on morphological evaluation, which is limited by subjectivity, static observation, and the difficulty of integrating heterogeneous clinical and biological data. In recent years, artificial intelligence has emerged as a decision-support tool in embryology, enabling the analysis of large datasets derived from embryo images, morphokinetic parameters, and clinical variables. This review summarizes current approaches to artificial intelligence in embryo selection, including models based on static images and time-lapse imaging data. Machine learning and deep learning techniques are discussed, including convolutional neural networks and spatiotemporal models. The evaluation of model performance is also examined, highlighting the clinical relevance of endpoints such as time to live birth compared with traditional outcome measures. Finally, ethical and clinical implications are considered, including issues related to transparency, responsibility, human oversight, and regulation. Artificial intelligence has the potential to improve embryo selection, although further validation and standardized implementation are needed before routine clinical use.

## 1. Introduction: The Bottleneck of Embryo Selection in IVF

Although advances in the field of assisted reproductive technologies are now considerable, the outcomes of in vitro fertilization are still not optimal. Improvements in ovarian stimulation protocols, in vitro fertilization procedures, embryo culture conditions, and the cryopreservation of gametes and embryos have certainly enhanced laboratory efficiency and expanded the therapeutic possibilities for success. Despite this, one of the main unresolved problems in clinical practice remains the ability to reliably identify the embryo with the highest developmental potential post-embryo transfer within a given cohort [1,2].

In many laboratories, embryo selection in routine ART cycles is still primarily based on conventional morphological assessment performed at predefined developmental time points. Within this framework, normal fertilization is confirmed by the presence of two pronuclei; during cleavage-stage development, embryos are evaluated according to cleavage dynamics, blastomere number, and morphological features; and, at the blastocyst stage (day 5/6), assessment is based on the degree of expansion and on the quality of the inner cell mass and trophectoderm, according to established grading systems such as the Gardner classification. This approach remains widely used because it is well established, practical, and fully integrated into the daily workflow of embryology laboratories. However, it has well-recognized limitations. Embryo development is a continuous and dynamic process, whereas conventional morphological assessment relies on static observations performed at predefined time points. As a result, potentially informative developmental events may occur outside these observation windows and therefore remain undetected by static assessment [1,3,4,5,6,7]. Another crucial aspect is subjectivity. Morphological grading depends largely on the embryologist’s interpretation of visual features, inevitably introducing a certain degree of variability [8]. When parameters such as cell symmetry, fragmentation, cleavage patterns, or blastocyst quality are assessed, this variability results in different evaluations. This, in turn, may lead to different embryo rankings, even when the same cohort is analyzed by experienced operators such as senior embryologists. All this does not represent merely a technical issue but rather a clinically relevant limitation, because embryo selection directly influences the choice of the best embryo to transfer and, consequently, treatment outcome [3,9].

The introduction of time-lapse systems has partially addressed these limitations. These systems allow continuous monitoring of embryos in culture and make it possible to obtain morphokinetic information that cannot be captured by conventional static assessment alone. This has expanded the amount of biological information available and has further highlighted the idea that the timing of embryo development may provide additional valuable indications of implantation potential. Different algorithms and scoring systems have been proposed, but their performance is not always reproducible across different laboratories, and their applicability may be influenced by culture conditions, annotation methods, technical variability, and patient-related factors [3,4,10].

Morphology, developmental kinetics, and clinical variables related to the patient or the treatment are all important sources of information, but they are often considered separately. This fragmentation represents one of the main reasons why embryo selection continues to be complex in clinical practice. Embryo competence and viability, in fact, do not depend on a single observable feature, and the clinical value of embryo ranking does not concern only the individual embryo but is placed within the broader context of treatment, including the characteristics of the entire embryo cohort [1,4,10,11].

For all these reasons, embryo selection still represents one of the main bottlenecks in IVF today. Although the currently available approaches are certainly useful in clinical practice, they remain limited by incomplete standardization, residual subjectivity, and a reduced ability to consistently integrate heterogeneous sources of biological and clinical information. It is precisely from these limitations that, in recent years, interest has grown in more objective, reproducible and integrative decision-support strategies [2,3].

Therefore, the aim of this narrative review is not only to summarize current artificial intelligence-based approaches for embryo assessment but also to critically examine their clinical relevance, methodological limitations, validation requirements, and implementation challenges. Particular attention is given to the distinction between algorithmic performance and true clinical utility, including whether AI-assisted embryo ranking can improve patient-centered outcomes such as live birth, cumulative treatment efficiency, and time to live birth.

**Figure 1:**
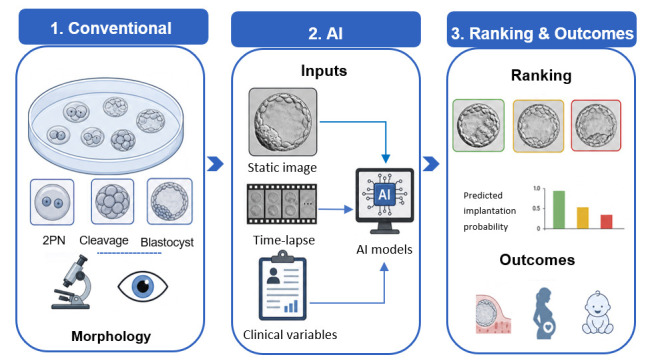
Schematic overview of embryo selection from conventional morphological assessment to AI-assisted ranking. Conventional evaluation is based on static embryo observations at key developmental stages, whereas AI-based approaches integrate static images, time-lapse information, and clinical variables to support embryo ranking and the prediction of clinically relevant outcomes.

A schematic overview of the transition from conventional embryo assessment to AI-assisted embryo ranking is shown in Figure 1.

## 2. Materials and Methods

This narrative review was conducted through a structured literature search of PubMed/MEDLINE, Web of Science, and Google Scholar for articles published in English up to April 2026. The search strategy was designed to identify studies addressing the application of artificial intelligence to embryo assessment, embryo ranking, and embryo selection in the context of assisted reproductive technology. Search terms included combinations of “artificial intelligence”, “machine learning”, “deep learning”, “neural network”, “convolutional neural network”, “computer vision”, “embryo selection”, “embryo assessment”, “embryo ranking”, “embryo grading”, “embryo viability”, “blastocyst image”, “static embryo image”, “time-lapse imaging”, “morphokinetics”, “in vitro fertilization”, “IVF”, and “assisted reproductive technology”.

Additional searches were performed using more specific terms related to clinically relevant outcomes and methodological aspects, including “embryo implantation”, “implantation potential”, “embryo ploidy”, “euploidy”, “aneuploidy”, “fetal heartbeat”, “clinical pregnancy”, “live birth”, “time to live birth”, “model validation”, “external validation”, “calibration”, “explainability”, “black-box model”, “clinical prediction model”, and “AI ethics”.

Reference lists of the selected articles and relevant reviews were manually screened to identify further studies of interest. Articles were included when they provided information relevant to the development, validation, interpretation, or clinical application of artificial intelligence models in embryo assessment and embryo selection. Particular attention was given to studies addressing model inputs, predicted outcomes, performance metrics, validation strategies, and clinical applicability. As this was a narrative review, the available evidence was summarized qualitatively, with the aim of highlighting current approaches, limitations, unresolved issues, and future perspectives in the use of artificial intelligence for embryo selection.

## 3. Why Artificial Intelligence Entered Embryo Selection

### 3.1. Artificial Intelligence in the Context of Embryology

In the field of reproductive medicine, artificial intelligence (AI) refers to computational approaches designed to analyze complex biomedical data and support clinical decision-making [12]. In IVF laboratories, AI can process large volumes of information derived from embryo images, morphokinetic parameters obtained from time-lapse systems, and patient clinical data [4,13,14]. By integrating these heterogeneous datasets, AI-based models aim to assist embryologists in predicting embryo viability, implantation potential, and treatment outcomes [13,14,15]. The application of AI in embryology seeks primarily to reduce the subjectivity associated with conventional embryo assessment. Traditional embryo evaluation relies largely on morphological criteria interpreted by embryologists, which may introduce inter- and intra-observer variability [5,16]. AI-driven approaches provide data-driven and standardized analyses, potentially improving the objectivity and reproducibility of embryo selection [13,14,16].

### 3.2. Machine Learning and Deep Learning in Embryology

Machine learning (ML) is a core component of artificial intelligence and includes algorithms capable of identifying patterns in data without explicit programming [12]. In reproductive medicine, conventional ML approaches rely on predefined variables, such as morphokinetic parameters, morphological scores, or clinical features, which are selected a priori by clinicians or researchers [4,5]. Deep learning (DL), a subset of ML, is based on artificial neural networks with multiple hidden layers that automatically extract relevant features from raw data [12]. In embryology, DL models—particularly convolutional neural networks (CNNs)—have demonstrated strong performance in the analysis of embryo images and time-lapse recordings, enabling the detection of complex visual and temporal patterns associated with embryo development and often outperforming traditional approaches based on manually defined features [13,15,16]. From a methodological perspective, AI models can be classified as interpretable (white-box) or non-interpretable (black-box) systems [17]. Interpretable models, such as linear models and decision trees, provide insight into the contribution of individual variables to the final prediction, whereas DL models are generally considered black-box systems due to their limited transparency [17]. Despite their high predictive performance, the reduced interpretability of DL approaches may limit their clinical applicability, underscoring the need to balance accuracy and explainability in AI-based embryo selection tools [15,17].

## 4. Learning from a Single Image: AI Models Based on Static Embryo Assessment

In the time-lapse setting, some AI models rely on a single selected frame rather than on the full video sequence. These static-image approaches, most often applied at the blastocyst stage, extract morphological features to predict clinically relevant outcomes, primarily embryo implantation potential and chromosomal status (ploidy) [13,14,18].

Several studies have investigated whether deep learning models, particularly convolutional neural networks (CNNs), can infer clinically relevant outcomes from morphological information contained in a single embryo image. One of the earliest and most influential examples is the study by Khosravi et al., who trained a CNN on more than 10,000 blastocyst images and reported an accuracy of 96.94% in distinguishing high-quality from low-quality embryos, showing that automated image analysis can identify morphological patterns that are difficult to quantify objectively by visual inspection alone [13]. In a more clinically oriented application, VerMilyea et al. developed the Life Whisperer system, a CNN-based model designed to classify embryos as viable or non-viable from static images. In independent blinded test sets, the model achieved a sensitivity of 70.1%, a specificity of 60.5%, and an overall accuracy of 64.3%, outperforming embryologist assessment in that setting [14]. More recently, the MAIA platform was prospectively tested in a routine clinical setting using static embryo images, further supporting the feasibility of image-based AI tools as embryologist-support systems [19].

Subsequent studies extended the scope of static-image AI beyond conventional morphology-based grading. Conversa et al. evaluated a deep learning model trained to predict fetal heartbeat after transfer of vitrified-warmed blastocysts from a single image, supporting the feasibility of extracting clinically relevant predictive information even in the absence of full developmental sequence analysis [20]. Chávez-Badiola et al. developed the Embryo Ranking Intelligent Classification Algorithm (ERICA), which used static blastocyst images to rank embryos according to predicted implantation potential and ploidy. In their study, ERICA ranked a euploid blastocyst first in 78.9% of cases and placed at least one euploid embryo within the top two positions in 94.7% of cases, outperforming both random classification and senior embryologists [18]. In addition, Bori, Meseguer, and colleagues explored a multimodal strategy combining blastocyst morphology with proteomic biomarkers. Their preliminary artificial intelligence models correctly classified embryos, leading or not leading to live birth, with accuracies of 100.0% for ANN1, 85.7% for ANN2, and 83.3% for ANN3, suggesting that integration of morphological and non-visual biological data may further improve embryo viability assessment [21].

Taken together, these studies show that the main value of static-image AI models lies not simply in automating conventional morphological evaluation but in extracting predictive information from embryo images for a range of clinically relevant outcomes, including embryo quality, viability, implantation-related potential, ploidy, fetal heartbeat, and live birth [13,14,18,20,21]. However, because these models rely on a single image, their performance remains intrinsically dependent on the timing and quality of image acquisition and does not account for the temporal dynamics of embryo development (Table 1).

## 5. Learning from Embryo Dynamics: Time-Lapse and Video-Based AI

One of the main advantages of time-lapse imaging is the ability to extract morphokinetic parameters, which describe the timing of key developmental events. Pioneering work by Meseguer et al. demonstrated that embryo implantation probability is closely linked to specific chronological milestones, identifying key predictive parameters such as the time of division to 5 cells (t5), the interval between the third and fourth cleavage (s2), and the duration of the second cell cycle (cc2). Furthermore, the observation of dysmorphic behaviors, such as abrupt division or multinucleation, provided a basis for multivariable selection models [4]. Building upon these clinical findings, Milewski et al. applied more advanced computational techniques, using artificial neural networks and principal component analysis (PCA) to quantify the predictive information within these morphokinetic datasets [22]. These approaches represent the first generation of AI-assisted embryo evaluation based on time-lapse imaging. Subsequent developments introduced deep learning models capable of analyzing entire time-lapse image sequences rather than predefined morphokinetic parameters. By processing large volumes of sequential embryo images, these models automatically learn spatial and temporal patterns associated with embryo competence.

More recent approaches have integrated advanced deep learning architectures, including convolutional neural networks combined with recurrent neural networks such as long short-term memory (LSTM) layers, to simultaneously analyze spatial and temporal information in embryo development. In this context, Paya et al. developed a spatiotemporal model specifically designed to classify embryo ploidy status using time-lapse videos from 10 to 115 h post-insemination. Their end-to-end approach utilized a CNN for spatial feature extraction and a bidirectional LSTM (or GRU) layer to analyze temporal dependencies, achieving a precision of 0.8205 for predicting euploidy and an overall accuracy of up to 0.7308 [23]. For instance, deep learning frameworks applied to time-lapse monitoring have achieved AUC values around 0.82 for predicting blastocyst formation and quality, highlighting the potential of temporal AI models to improve embryo selection [24].

Additional studies have further explored AI models using time-lapse datasets to predict clinically relevant outcomes. For example, Maekawa et al. developed an AI system trained on time-lapse images to predict both implantation potential and embryo euploidy, demonstrating that developmental dynamics may contain information related not only to embryo viability but also to chromosomal integrity [25]. Similarly, recent deep learning models analyzing time-lapse videos (such as those by Boucret et al.) have been trained on datasets of embryos with known implantation outcomes, enabling the identification of dynamic patterns associated with successful pregnancy [26].

Compared with approaches based solely on static images, video AI models offer several advantages. By capturing the continuous progression of embryo development, these systems provide information on cleavage dynamics, cell cycle synchronization, and developmental irregularities that cannot be inferred from a single image. As a result, dynamic models may detect subtle temporal patterns associated with embryo competence, potentially improving predictive performance and reducing subjectivity in embryo evaluation.

However, the use of time-lapse data also introduces technical and operational challenges. Time-lapse imaging generates extremely large datasets, requiring substantial computational resources for storage, processing, and model training. In addition, the development of reliable AI models depends on access to high-quality annotated video datasets, which remain limited in many clinical settings. Another important limitation is the dependence on specific time-lapse incubator systems, which may vary between laboratories and affect the generalizability of trained models. As highlighted in recent systematic reviews, these challenges regarding standardization and computational demand remain significant hurdles for the universal clinical implementation of automated grading systems [27].

The main characteristics of representative artificial intelligence models based on time-lapse imaging and embryo developmental dynamics are summarized in Table 2.

## 6. How to Evaluate AI Models: Metrics and Clinical Relevance

The performance of AI models for embryo selection should be assessed using metrics that reflect both predictive accuracy and clinical applicability. The TRIPOD + AI guideline [28] outlines how the performance of clinical prediction models should be reported, emphasizing three main aspects: model discrimination, calibration, and clinical usefulness. Most studies assessing AI algorithms in IVF rely on conventional performance metrics that capture different aspects of model performance. The area under the receiver operating characteristic curve (AUC) measures discrimination, that is, the ability of a model to distinguish between embryos with and without the outcome of interest across all possible classification thresholds [28,29]. Accuracy reflects the overall proportion of correctly classified cases, whereas sensitivity and specificity indicate the proportions of positive and negative cases correctly identified, respectively [30,31]. Accordingly, AUC, accuracy, sensitivity, and specificity remain among the most frequently reported metrics in studies evaluating AI-based embryo selection models [14,32,33]. These measures primarily quantify the ability of a model to discriminate between embryos associated with different outcomes, typically positive implantation or live birth. Discrimination metrics such as the AUC are widely used to assess the performance of AI models and to compare different algorithms or embryo ranking systems, and they remain among the most frequently reported indicators in the embryo selection literature [34]. Moreover, comparisons based on AUC can be misleading when models are evaluated across studies involving different embryo populations, outcome definitions, or label distributions, as these factors can substantially influence performance estimates and introduce bias in the interpretation of relative algorithm performance [35,36].

Nevertheless, in the clinical IVF setting, the key parameter is not to identify the absolute probability of success for an individual embryo across the entire population but rather to determine which embryo among those within the same cohort should be transferred first. In fact, one of the most significant challenges worldwide is the discontinuation of treatment by couples after one or more unsuccessful IVF attempts. This phenomenon, commonly referred to as treatment dropout, represents a major limitation in assisted reproduction because many couples interrupt the treatment pathway before completing the number of cycles required to achieve a high cumulative probability of live birth [37]. The causes of this phenomenon are complex and multifactorial, with psychological and socioeconomic factors being some of the most common ones reported in scientific literature [38].

For this reason, increasing attention has been directed toward analytical approaches that assess AI model performance within the same treatment cycle. In this context, the relevant question becomes whether an algorithm can correctly rank embryos belonging to the same cohort according to their probability of leading to a live birth. To address this issue, the concept of time to live birth has recently been proposed as a clinically relevant endpoint. Time to live birth (TTLB) is defined as the time or number of embryo transfer attempts required to achieve a live birth within a given treatment pathway when embryos are transferred according to a specific ranking or selection strategy. This endpoint provides a more accurate representation of how efficiently a selection method leads to the first live birth.

Particularly, in a recent publication, Bori et al. proposed a novel methodological approach to evaluate the TTLB and the clinical utility of embryo selection algorithms by considering all usable embryos within a treatment cycle, including those transferred and not transferred [2]. A multiple imputation approach by chained equations (MICE) was used to estimate the outcomes of non-transferred embryos [39] and applied to a large dataset. TTLB was then compared between AI-based embryo ranking and conventional morphological grading, showing a 6.1% shorter estimated TTLB with AI-guided selection [2].

Importantly, the distinction between algorithmic performance and clinical utility has also been highlighted by recent randomized evidence. In a randomized, double-blind non-inferiority trial comparing iDAScore-based deep learning embryo selection with standard morphology-based assessment, Illingworth et al. reported similar clinical pregnancy rates between groups, but non-inferiority of the deep learning strategy was not demonstrated [40].

## 7. Discussion

Artificial intelligence is increasingly being explored as a decision-support tool in embryo selection because it addresses some of the main limitations of conventional assessment, including subjectivity, inter-observer variability, and the difficulty of integrating heterogeneous sources of information. As discussed in the previous sections, AI models have been developed using both static embryo images and time-lapse data, with the aim of predicting clinically relevant outcomes such as embryo quality, implantation potential, ploidy, fetal heartbeat, and live birth [13,14,18,20,21,22,23,24,25,26]. These approaches differ in terms of input data, model architecture, and target outcomes, but collectively they show that embryo evaluation can be enhanced by computational methods capable of identifying complex visual and temporal patterns beyond conventional manual assessment [4,13,22,23].

At the same time, the current literature also highlights important limitations. The performance of AI models is often reported using conventional discrimination and classification metrics, such as AUC, accuracy, sensitivity, and specificity, which are useful but do not necessarily reflect the true clinical value of a model in routine IVF practice [14,28,29,30,31,32,33]. In embryo selection, the clinically relevant question is not only whether a model performs well at the population level but also whether it improves ranking decisions within the same cohort and, ultimately, shortens the path to live birth. In this regard, recently proposed approaches such as time to live birth may provide a more clinically meaningful framework for evaluating AI-assisted embryo ranking strategies [2].

Another major issue is generalizability. AI models in reproductive medicine are often developed and tested in specific laboratory settings, using locally generated datasets, proprietary imaging systems, and center-specific annotation practices. This may limit reproducibility across clinics and reduce confidence in the transportability of model performance [27,35]. Furthermore, many currently available models remain insufficiently characterized in terms of external validation, calibration, and prospective clinical impact, all of which are essential for responsible implementation in real-world IVF settings [28,41,42].

The implementation of artificial intelligence in embryo selection requires consideration of several methodological, clinical, and ethical domains. Beyond predictive performance, future studies should address issues related to validation, reporting quality, clinical endpoints, interpretability, laboratory integration, and patient trust. These key aspects are summarized in Table 3 [28,41,42,43,44,45,46,47,48,49,50,51].

Importantly, the limitations summarized in Table 3 indicate that the clinical value of AI in embryo selection cannot be inferred from predictive performance alone. A model with good discrimination may still have limited clinical utility if it has not been externally validated, if its probability estimates are poorly calibrated, or if the outcome used for training does not correspond to a meaningful patient-centered endpoint [28]. This is particularly relevant in IVF, where embryo selection is not only a binary prediction task but also a ranking process within a specific embryo cohort [35]. For this reason, future studies should move beyond isolated performance metrics and evaluate whether AI-assisted ranking can improve the efficiency of treatment, reduce unnecessary embryo transfers, and shorten the time to live birth [2,43].

In this context, AI-assisted embryo selection should be approached with the same level of scrutiny applied to other reproductive medicine add-ons, requiring evidence of safety, effectiveness, and clinically meaningful benefit before widespread routine implementation [52].

Another important consideration is the transition from model development to clinical implementation. Many AI systems are developed under controlled research conditions, whereas routine IVF laboratories differ in terms of incubator platforms, culture protocols, image acquisition settings, embryo annotation practices, patient characteristics, and clinical policies [27]. These differences may substantially affect model performance and limit generalizability [35]. Therefore, before AI-based embryo selection tools are incorporated into routine practice, they should be evaluated in independent clinical settings and ideally within prospective implementation studies [28]. Such studies should assess not only predictive accuracy but also workflow integration, human–AI interaction, embryologist acceptance, patient communication, and the potential impact on clinical decision-making [41,42,51].

These methodological challenges are closely linked to ethical and professional considerations. In embryo selection, AI does not operate in a neutral technical space but within a high-stakes clinical context in which decisions directly influence treatment strategy and patient expectations. For this reason, predictive performance alone is not sufficient to justify clinical adoption. Transparency, explainability, accountability, fairness, and appropriate human oversight remain essential requirements for the responsible use of AI in IVF [41,42,43,44,45,46,47,48,49,50]. These requirements also extend to informed patient communication, since couples should be made aware of the role of AI in embryo assessment, the type of data used to generate predictions, and the limits of algorithmic output in the clinical decision-making process. In addition, the risk of automation bias should be carefully considered, as embryologists and clinicians may be inclined to over-rely on AI-generated rankings, particularly when model predictions appear highly accurate but remain only partially interpretable. Another important issue concerns fairness and dataset representativeness. Because many AI models are developed on retrospective, single-center, or laboratory-specific datasets, their performance may not be equally reliable across different patient populations, clinical settings, or laboratory conditions, with the consequent risk of embedding or amplifying pre-existing biases. Data governance also remains a central ethical concern, given that AI-assisted embryo selection may involve the large-scale use of sensitive reproductive, imaging, and potentially genetic data, requiring robust safeguards for privacy, traceability, and responsible data sharing. For these reasons, the clinical integration of AI in embryo selection should remain grounded in meaningful human oversight, transparent validation, and clearly defined professional responsibility so that algorithmic support strengthens rather than replaces expert clinical judgment. Importantly, current evidence does not support replacing embryologist judgment with algorithmic output; rather, AI should be integrated as a support tool embedded within expert clinical and laboratory practice [44,45,47].

Overall, the available evidence suggests that AI has considerable potential to improve embryo selection, particularly by enabling more standardized, data-driven, and scalable evaluation strategies. However, its current clinical role should still be considered supportive rather than definitive. Further progress will depend not only on technical refinement but also on robust validation, transparent reporting, and careful alignment between algorithmic development and the ethical, clinical, and organizational realities of reproductive medicine [2,28,41,42,43,44,45,46,47,48,49,50].

## 8. Future Directions

Future developments in this field will likely move beyond isolated prediction models toward a more integrated concept of the intelligent IVF laboratory. In this setting, AI could combine multiple sources of information generated throughout the laboratory workflow, including static and time-lapse imaging, morphokinetic annotation, embryology records, patient and cycle characteristics, laboratory environment data, and treatment outcomes. The integration of these heterogeneous data streams may allow the development of more comprehensive and clinically useful prediction models, better reflecting the complexity of embryo competence and treatment success [2,28].

In parallel, AI may also contribute to laboratory quality control. Potential applications include automated image annotation, consistency checks in embryo grading, monitoring of incubator and workflow parameters, detection of deviations in laboratory performance, and support for traceability and auditing processes. However, these developments should be pursued within clearly defined ethical and regulatory boundaries, with particular attention to data quality, bias control, transparency, human supervision, and patient trust [41,42,43,44,45,46,47,48,49,50].

In conclusion, future progress in AI-assisted embryo selection should not be defined solely by increasingly accurate algorithms but by the development of clinically meaningful, ethically sound, and well-governed systems that support both embryo selection and overall laboratory quality within routine IVF practice.

## 9. Conclusions

Artificial intelligence represents one of the most promising developments in embryo selection, offering the possibility of more objective, reproducible, and data-driven assessment. Current evidence suggests that AI models can extract clinically relevant information from static embryo images, time-lapse imaging, morphokinetic data, and multimodal datasets. However, the available literature also shows important limitations, including heterogeneous endpoints, limited external validation, variable reporting quality, and uncertainty regarding clinical implementation.

At present, AI should be considered a support tool rather than an autonomous decision-maker in embryo selection. Its clinical value will depend on whether future studies can demonstrate not only good predictive performance but also improved embryo ranking, better workflow integration, and meaningful benefits for patients. Robust validation, transparent reporting, ethical governance, and continued embryologist oversight will be essential before AI-based embryo selection can be fully integrated into routine IVF practice.

## Figures and Tables

**Table 1:** Main characteristics of the static-image artificial intelligence models included in this review, including input type, model architecture, predicted outcome, and reported performance metrics. AI, artificial intelligence; ANN, artificial neural network; CNN, convolutional neural network.

Study	Input	Model	Main Predicted Outcome	Reported Performance
Khosravi et al. [13]	Single blastocyst image	CNN	Embryo quality classification	Accuracy: 96.94%
VerMilyea et al. [14]	Static embryo image	CNN (Life Whisperer)	Viable vs. non-viable embryo classification	Sensitivity: 70.1%; Specificity: 60.5%; Accuracy: 64.3%
Conversa et al. [20]	Single image of vitrified-warmed blastocyst	Deep learning model	Fetal heartbeat prediction	Predictive ability reported; metric to specify from original article
Chávez-Badiola et al. [18]	Static blastocyst image	ERICA	Embryo ranking for implantation potential and ploidy	Euploid embryo ranked first: 78.9%; at least one euploid embryo in top two: 94.7%
Bori et al. [21]	Blastocyst morphology + proteomic profile	ANN	Live birth classification	ANN1: 100.0%; ANN2: 85.7%; ANN3: 83.3%

**Table 2:** Main characteristics of time-lapse and video-based artificial intelligence models included in this review.

Study	Input	Model	Main Predicted Outcome	Reported Performance
Milewski et al. [22]	Time-lapse morphokinetic parameters, fragmentation, multinucleation, blastomere evenness, and female age	ANN combined with PCA	Embryo implantation potential	AUC: 0.75; validation set AUC: 0.71
Liao et al. [24]	Time-lapse videos from the first three days of embryo development	Deep learning ensemble models integrating DenseNet201, LSTM, and gradient boosting classifiers	Blastocyst formation and usable blastocyst prediction	STEM: accuracy 78.2%, AUC 0.82; STEM+: accuracy 71.9%, AUC 0.79
Paya et al. [23]	Time-lapse videos from 10 to 115 h post-insemination	Spatiotemporal deep learning model combining CNN-based feature extraction with recurrent layers	Embryo ploidy status	Best model: precision 0.8205 for euploidy prediction; sensitivity 0.6957; specificity 0.7813; accuracy 0.7308
Boucret et al. [26]	Time-lapse videos of matched high-quality embryos with known implantation data	Self-supervised contrastive learning, Siamese neural network, and XGBoost prediction model	Implantation outcome among morphologically good-quality embryos	AUC: 0.64 without transfer-history information; AUC: 0.57 when using the previous transfer outcome from the same cycle
Maekawa et al. [25]	Time-lapse videos and maternal age	Spatiotemporal convolutional neural network	Clinical pregnancy and embryo ploidy status	Pregnancy model AUROC: 0.799 validation set, 0.717 internal test set, 0.746 external test set; ploidy model AUROC: 0.802 validation set, 0.738 internal test set, 0.759 external test set

**Table 3:** Key challenges and recommended directions for the clinical implementation of artificial intelligence in embryo selection. AI, artificial intelligence; IVF, in vitro fertilization; TTLB, time to live birth.

Domain	Critical Issue	Recommended Direction
External validity	Many AI models are developed on retrospective or single-center datasets, limiting their generalizability across IVF laboratories	Validate models in independent, multicenter populations before routine clinical use [27,28,35]
Performance assessment	Reported performance often focuses on AUC, accuracy, sensitivity, and specificity, while calibration and clinical utility are less consistently addressed	Evaluate discrimination together with calibration, clinical usefulness, and transparent reporting standards [28,41,42,51]
Clinical endpoints	Outcomes such as embryo quality, implantation, ploidy, or clinical pregnancy may not fully reflect the patient-centered goal of treatment	Prioritize live birth and TTLB as clinically meaningful endpoints for embryo-ranking strategies [2,43]
Interpretability	Deep learning models may provide limited insight into how predictions are generated	Develop interpretable outputs that support embryologist judgment without replacing clinical responsibility [17,44,45]
Clinical and ethical governance	Issues related to accountability, fairness, patient communication, data protection, and regulation remain incompletely defined	Implement AI within transparent governance frameworks, preserving human oversight and patient trust [41,42,43,44,45,46,47,48,49,50]

## Data Availability

No new data were created.

## Abbreviations

The following abbreviations are used in this manuscript:

AI	Artificial Intelligence
ANN	Artificial Neural Network
ART	Assisted Reproductive Technologies
AUC	Area Under the Receiver Operating Characteristic Curve
CNN	Convolutional Neural Network
CONSORT AI	Consolidated Standards of Reporting Trials Artificial Intelligence extension
DL	Deep Learning
ERICA	Embryo Ranking Intelligent Classification Algorithm
GRU	Gated Recurrent Unit
IVF	In Vitro Fertilization
LSTM	Long Short-Term Memory
MICE	Multiple Imputation by Chained Equation
ML	Machine Learning
PCA	Principal Component Analysis
SPIRIT AI	Standard Protocol Items Recommendations for Interventional Trials Artificial Intelligence extension
TRIPOD AI	Transparent Reporting of a multivariable prediction model for Individual Prognosis or Diagnosis Artificial Intelligence extension
TTLB	Time To Live Birth
